# Features of Mitochondrial Dynamics in Different Brain Parts of Patients with Alzheimer’s Disease

**DOI:** 10.3390/ijms27010107

**Published:** 2025-12-22

**Authors:** Vladimir S. Sukhorukov, Tatiana I. Baranich, Olga V. Velts, Dmitry N. Voronkov, Ekaterina V. Shcherbak, Dmitry S. Lazarev, Dmitry A. Kharlamov, Alexandr P. Raksha

**Affiliations:** 1 Laboratory of Neuromorphology, Russian Center of Neurology and Neurosciences, 125367 Moscow, Russia; vsukhorukov@gmail.com (V.S.S.);; 2 Department of Morphology, Pirogov Russian National Research Medical University, 117513 Moscow, Russia; 3 Pirogov City Clinical Hospital No. 1, 119049 Moscow, Russia; 4 Voyno-Yasenetsky Scientific and Practical Center of Specialized Medical Care for Children, 119620 Moscow, Russia

**Keywords:** Alzheimer’s disease, aging, neuron, mitochondrial dynamics, amyloid, hippocampus

## Abstract

A number of recent studies have demonstrated the importance of investigating mitochondrial dynamics mechanisms in Alzheimer’s disease (AD). This study involved an immunohistochemical analysis of mitochondrial dynamics markers Opa-1, Mfn-2, and Drp-1 in neurons of the middle frontal gyrus, anterior cingulate gyrus, head of the caudate nucleus, hippocampus, and inferior parietal lobule using autopsy material from AD patients. The comparison was made with similar brain regions in autopsy material from elderly and young patients. Additionally, changes in these markers in AD were correlated with the distribution of pathological amyloid aggregates in the corresponding regions. Our findings suggest specific features of metabolic process alterations in neurons in AD, which are not limited to a single brain region but involve multiple areas and exhibit a uniform pattern. Our data are significant for understanding the pathogenesis of this disease and aging in general, and may serve as a basis for developing targeted therapy for AD.

## 1. Introduction

Alzheimer’s disease (AD) is a heterogeneous, multifactorial neurodegenerative disorder that most commonly affects individuals over 65 years of age. According to the WHO, Alzheimer’s disease represents the most prevalent form of dementia, accounting for approximately 60–70% of patients with this condition. WHO estimates indicate that over 55 million people worldwide have dementia (8.1% of women and 5.4% of men aged over 65). This figure is projected to rise to 78 million by 2030 and 139 million by 2050. As evidenced, the development of pharmacological therapies for AD faces numerous challenges associated with drug inefficacy and atypical patient responses to these agents [1,2,3], necessitating comprehensive investigation of fundamental biological processes underlying AD progression to advance pathogenetic understanding and identify novel targets for targeted therapies.

A key feature of AD is the heterogeneity in the manifestation of pathological processes, their onset, and progression across different regions of the brain. Anatomical and histological studies of AD patients’ brains have demonstrated that neurodegenerative processes originate in layer II of the entorhinal cortex and gradually spread to the hippocampus, temporal cortex, frontoparietal cortex, and subcortical nuclei [4]. At later stages, the disruption of connectivity between the hippocampus and other brain regions results in cognitive impairments [5,6,7]. It has been hypothesized that the disconnection between the entorhinal cortex and hippocampus underlies memory processing deficits [8]. Notably, AD is associated with a significant reduction in the volume of the caudate nucleus, which is considered an early diagnostic marker of the disease [9]. Recent studies suggest that caudate nucleus atrophy contributes to memory and learning impairments, apathy, emotional dysregulation, and motor coordination deficits in AD [9,10,11]. The involvement of the inferior parietal lobule and anterior cingulate gyrus in the pathological process leads to diminished capacity for empathy and self-awareness in AD patients, consequently reducing social interaction [12].

The key pathomorphological changes in AD are known to be neurofibrillary tangles (aggregates of phosphorylated tau protein in neuronal perikarya) and amyloid plaques. However, the detailed mechanism of AD remains unexplored, with numerous studies demonstrating the fundamental role of mitochondria in its pathogenesis [13,14,15,16].

A number of recent studies have demonstrated the importance of investigating mitochondrial dynamics mechanisms in AD. The term “mitochondrial dynamics” primarily encompasses mitochondrial fission and fusion, which affect their spatial distribution, size, and quantity. Mitofusins (Mfn-1 and Mfn-2), proteins of the outer mitochondrial membrane, participate in the GTP-dependent mitochondrial fusion process [17] by regulating the fusion of outer membranes between two mitochondria. Additionally, Mfn-2 facilitates the formation of mitochondria-associated membrane (MAM) complexes with the endoplasmic reticulum [18,19]. The inner mitochondrial membranes undergo fusion under the control of the cytoplasmic protein Opa-1 (Optic Atrophy-1) [20]. Conversely, the cytoplasmic GTPase Drp-1 (Dynamin-related protein 1) serves as the key regulator of mitochondrial fission: following the recruitment of Drp-1 to the outer mitochondrial membrane, dimers and oligomers of Drp-1 assemble into a ring-like structure that mediates the division of both outer and inner mitochondrial membranes. Mitochondrial proliferation may serve to maintain their quantity and facilitate the clearance of defective organelle fragments [21].

Numerous studies indicate that impaired mitochondrial dynamics constitutes a significant component of neurodegeneration in AD. The study by Manczak et al. [22] demonstrated that beta-amyloid accumulation plays a critical role in disrupting mitochondrial dynamics and inhibiting mitochondrial biogenesis. Specifically, an examination of the hippocampus in APP transgenic mice revealed elevated Drp-1 levels alongside reduced levels of key fusion markers Mfn-1, Mfn-2, and Opa-1. Increased levels of Mfn1/2 and Opa-1 were observed, leading to a shift in mitochondrial dynamic balance toward fusion in vitro within primary hippocampal neurons of rats overexpressing wild-type tau protein. Concurrently, knockdown of Mfn1/2 facilitated the restoration of normal mitochondrial and neuronal function following mitochondrial dynamic imbalance, whereas Opa-1 knockdown did not produce this effect [23]. Additionally, the study by Xu et al. revealed elevated Mfn-2 levels in vivo within hippocampal neurons of 3- and 6-month-old APP mice modeling Alzheimer’s disease progression. At 3 months of age, these mice exhibited increased expression of Opa-1 and Drp-1 proteins, whereas by 6 months, their levels decreased compared to the control group [24]. Thus, existing data on the nature of mitochondrial dynamic alterations in AD remain contradictory [25] and require further clarification across distinct brain regions, which became the objective of this study.

## 2. Results

### 2.1. Middle Frontal Gyrus

**Figure 1:**
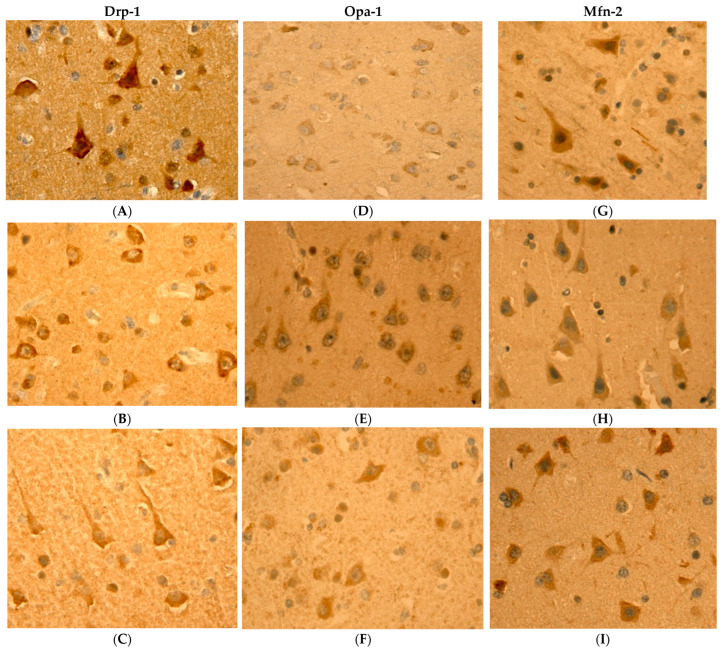
Example of performed immunohistochemical staining. Neurons in lamina 5 of middle frontal gyrus. (**A**) Drp-1 staining in AD; (**B**) Drp-1 staining in normal aging; (**C**) Drp-1 staining in control group; (**D**) Opa-1 staining in AD; (**E**) Opa-1 staining in normal aging; (**F**) Opa-1 staining in control group; (**G**) Mfn-2 staining in AD; (**H**) Mfn-2 staining in normal aging; (**I**) Mfn-2 staining in control group.

The middle frontal gyrus exhibited the most pronounced development of layers 3 and 5, containing large pyramidal neurons characteristic of these layers. Immunohistochemical staining for the Opa-1 marker was observed in the subplasmalemmal region of pyramidal neurons in layers 3 and 5 of the middle frontal gyrus in both control (young) groups and cases of physiological and pathological aging. Immunohistochemical staining for the Drp-1 marker demonstrated intense expression without visible compartmentalization across all study groups. The distribution pattern of the Mfn-2 marker showed relative uniformity in the perikarya of neurons in layers 3 and 5 (Figure 1).

**Figure 2:**
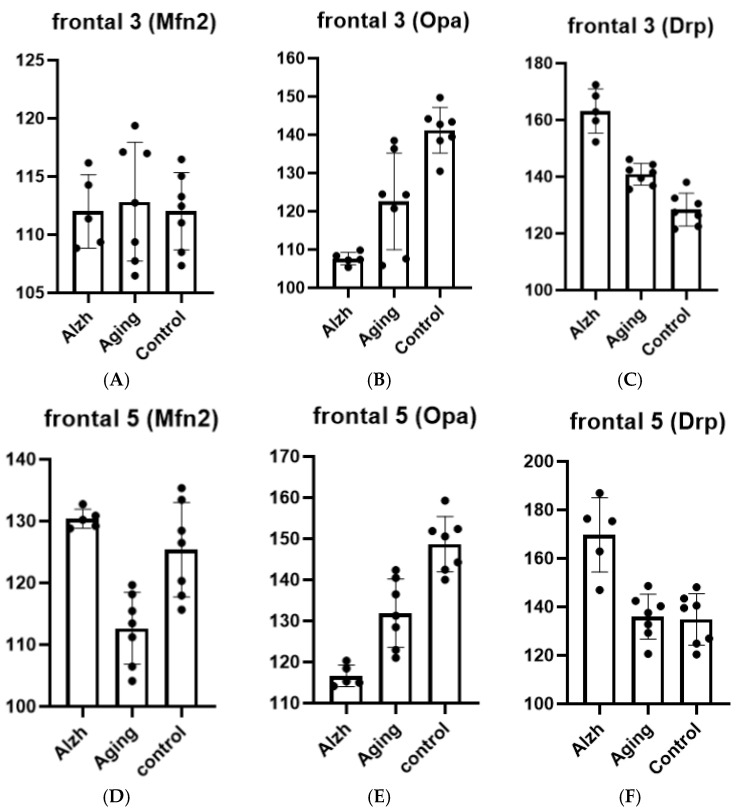
Results of morphometric analysis for immunohistochemical distribution of Mfn-2, Opa-1, and Drp-1 markers in neurons of layers 3 and 5 of the middle frontal gyrus. (**A**)—change in Mfn-2 marker level in layer 3 neurons of the middle frontal gyrus; (**B**)—change in Opa-1 marker level in layer 3 neurons of the middle frontal gyrus; (**C**)—change in Drp-1 marker level in layer 3 neurons of the middle frontal gyrus; (**D**)–change in Mfn-2 marker level in layer 5 neurons of the middle frontal gyrus; (**E**)–change in Opa-1 marker level in layer 5 neurons of the middle frontal gyrus; (**F**)—change in Drp-1 marker level in layer 5 neurons of the middle frontal gyrus. Ordinate axis—arbitary unit.

In aging cases, layer 3 of the frontal cortex displayed increased Drp-1 levels (*p* = 0.0009), decreased Opa-1 levels (*p* = 0.0009), and unchanged Mfn-2 levels compared to young control cases. In AD, layer 3 of the frontal cortex demonstrated further elevation of Drp-1 levels (*p* ≤ 0.0001) and reduction in Opa-1 levels (*p* = 0.0092), with no alterations in Mfn-2 levels compared to aging cases (Figure 2A–C).

In aging cases, layer 5 of the frontal cortex revealed decreased Mfn-2 (*p* = 0.0010) and Opa-1 levels (*p* = 0.0002) with stable Drp-1 levels compared to young controls, whereas AD cases showed increased Drp-1 (*p* = 0.0001) and Mfn-2 levels (*p* = 0.0001) accompanied by reduced Opa-1 levels (*p* = 0.0013) relative to aging cases (Figure 2D–F).

### 2.2. Inferior Parietal Lobule

The inferior parietal lobule is characterized by the marked prominence of layers 3 and 5, which predominantly contain pyramidal neurons, as well as layers 2 and 4 typical of the granular cortex, containing various types of non-pyramidal neurons. Immunohistochemical staining for all three investigated markers in perikarya demonstrated diffuse and relatively homogeneous distribution with poorly expressed compartmentalization of marker localization across all neuronal types in the aforementioned layers.

**Figure 3:**
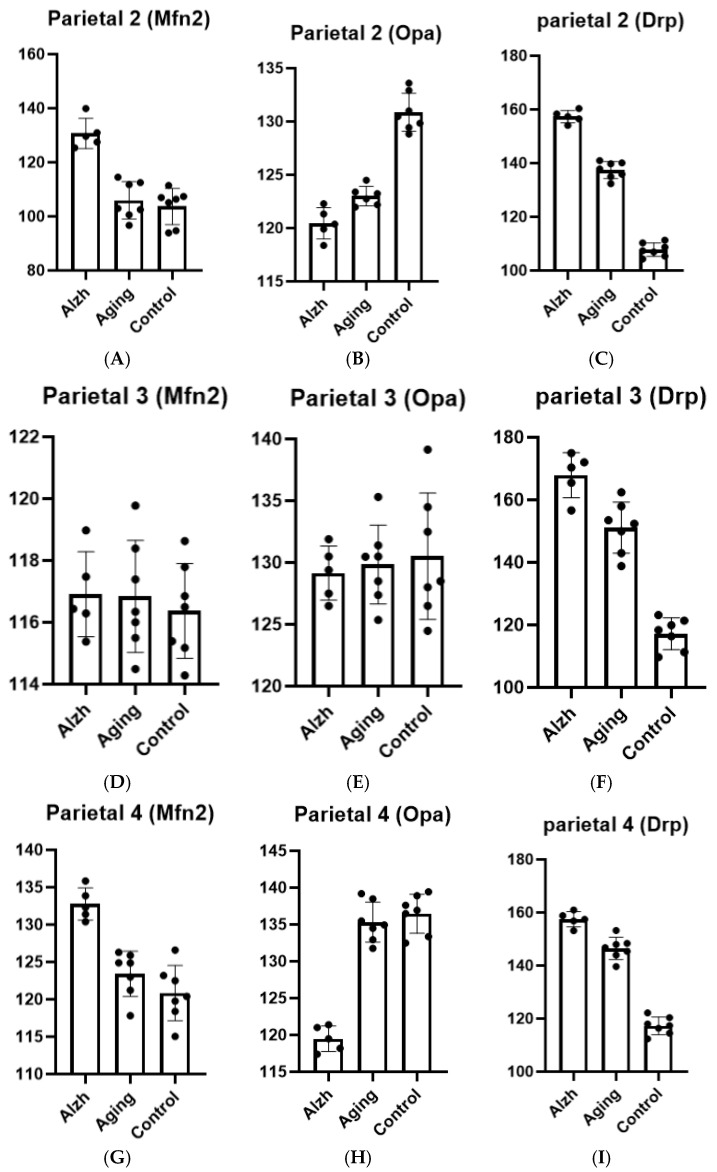

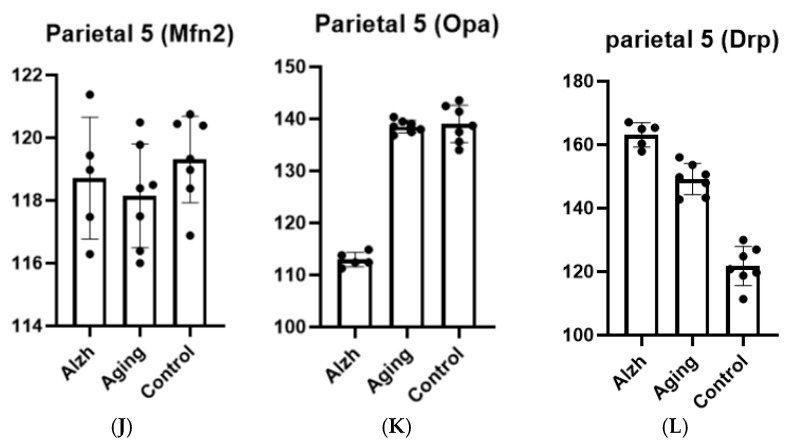
(**A**–**C**) Results of morphometric analysis of immunohistochemical distribution of Mfn-2 (**A**), Opa-1 (**B**), and Drp-1 (**C**) markers in layer 2 neurons of the inferior parietal lobule. (**D**–**F**) Results of morphometric analysis of immunohistochemical distribution of Mfn-2 (**D**), Opa-1 **(E),** and Drp-1 **(F)** markers in layer 3 neurons of the inferior parietal lobule. (**G**–**I**) Results of morphometric analysis of immunohistochemical distribution of Mfn-2 (**G**), Opa-1 (**H**), and Drp-1 (**I**) markers in layer 4 neurons of the inferior parietal lobule. (**J**–**L**) Results of morphometric analysis of immunohistochemical distribution of Mfn-2 (**J**), Opa-1 (**K**), and Drp-1 (**L**) markers in layer 5 neurons of the inferior parietal lobule. Ordinate axis—arbitary unit.

In layer 2 of the inferior parietal lobule, aging is associated with increased Drp-1 levels (*p* ≤ 0.0001), decreased Opa-1 levels (*p* ≤ 0.0001), and unchanged Mfn-2 levels compared to controls, whereas AD demonstrates increased Drp-1 (*p* ≤ 0.0001) and Mfn-2 (*p* ≤ 0.0001) levels alongside decreased Opa-1 levels (*p* = 0.0115) (Figure 3A–C).

In layer 3 of the inferior parietal lobule, no significant changes in Opa-1 or Mfn-2 levels were observed in either aging or AD, while Drp-1 levels increased in both aging (*p* < 0.0001) and AD (*p* = 0.0008) (Figure 3D–F).

In layer 4 of the inferior parietal lobule, no changes in Opa-1 and Mfn-2 marker levels were observed during aging compared to controls, while an increase in Drp-1 marker levels was noted (*p* ≤ 0.0001). In AD, in addition to elevated Drp-1 levels (*p* ≤ 0.0001) and reduced Opa-1 levels (*p* ≤ 0.0001), an increase in Mfn-2 levels was also detected (*p* = 0.0001) (Figure 3G–I).

In layer 5 of the inferior parietal lobule, no changes in Opa-1 and Mfn-2 marker levels were observed during aging compared to controls, with an increase in Drp-1 marker levels noted (*p* ≤ 0.0001). However, in AD, Drp-1 marker levels increased (*p* = 0.0003) and Opa-1 levels decreased (*p* = 0.0115), while Mfn-2 marker levels remained unchanged (*p* ≤ 0.0001) (Figure 3J–L).

### 2.3. Anterior Cingulate Cortex

The anterior cingulate cortex exhibited the most pronounced development of layers 3 and 5, containing large pyramidal neurons characteristic of these layers. The immunohistochemical staining pattern of Opa-1 marker was similar to that observed in the middle frontal gyrus: staining was pronounced in the subplasmalemmal region of pyramidal neurons in layers 3 and 5 of the anterior cingulate gyrus in both control (young) cases and cases of physiological and pathological aging. The immunohistochemical staining of Drp-1 marker appeared more diffuse in both control (young) cases and aging groups, while the distribution of Mfn-2 marker remained relatively homogeneous in neuronal perikarya of layers 3 and 5.

**Figure 4:**
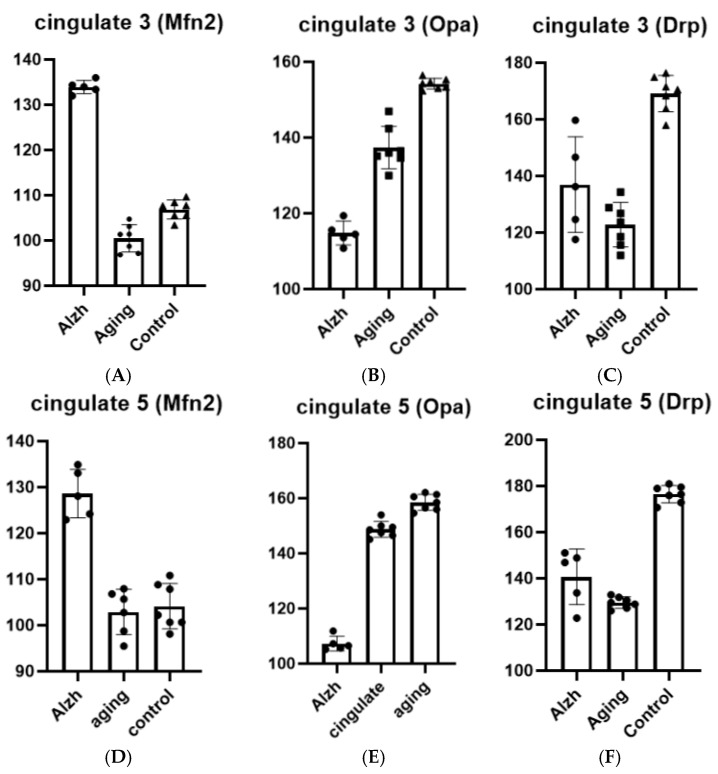
Results of morphometric analysis for immunohistochemical distribution of Mfn-2, Opa-1, and Drp-1 markers in neurons of layers 3 and 5 of the anterior cingulate cortex. (**A**)—change in Mfn-2 marker level in layer 3 neurons of the anterior cingulate cortex; (**B**)—change in Opa-1 marker level in layer 3 neurons of the anterior cingulate cortex; (**C**)—change in Drp-1 marker level in layer 3 neurons of the anterior cingulate cortex; (**D**)—change in Mfn-2 marker level in layer 5 neurons of the anterior cingulate cortex; (**E**)—change in Opa-1 marker level in layer 5 neurons of the anterior cingulate cortex; (**F**)—change in Drp-1 marker level in layer 5 neurons of the anterior cingulate cortex. Ordinate axis—arbitary unit.

In aging cases, layer 3 of the cingulate gyrus showed significant decreases in Opa-1 (*p* ≤ 0.0001), Mfn-2 (*p* = 0.0001), and Drp-1 (*p* ≤ 0.0001) levels compared to controls (young). In AD cases, layer 3 of the cingulate gyrus demonstrated increased Drp-1 (*p* = 0.0350) and Mfn-2 (*p* ≤ 0.0001) levels along with decreased Opa-1 levels (*p* ≤ 0.0001) compared to aging cases (Figure 4A–C).

In layer 5 of the cingulate gyrus, aging was associated with decreased Opa-1 (*p* ≤ 0.0001) and Drp-1 (*p* ≤ 0.0001) levels, while Mfn-2 levels remained unchanged compared to controls. In AD cases, layer 5 exhibited increased Drp-1 (*p* = 0.0108) and Mfn-2 (*p* ≤ 0.0001) levels accompanied by decreased Opa-1 levels (*p* ≤ 0.0001) compared to aging cases (Figure 4D–F).

### 2.4. Hippocampus

Pyramidal neurons in CA1 and CA2 regions were evaluated. The CA2 region is characterized by a higher cell density compared to CA1. Nevertheless, the immunohistochemical distribution of all three studied markers in perikarya of pyramidal neurons showed relative homogeneity.

**Figure 5:**
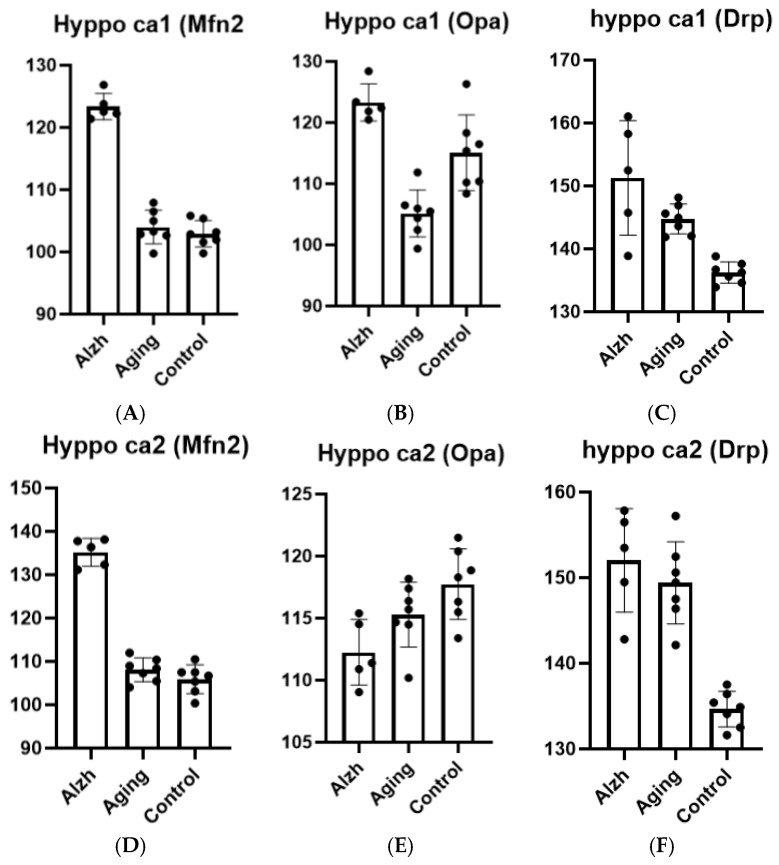
Results of morphometric analysis for immunohistochemical distribution of mitochondrial dynamics markers in CA1 hippocampal neurons: Mfn-2 (**A**), Opa-1 (**B**), and Drp-1 (**C**) markers; in CA2 hippocampal neurons: Mfn-2 (**D**), Opa-1 (**E**), and Drp-1 (**F**) markers. Ordinate axis—arbitary unit.

Hippocampal aging in CA1 demonstrates the following changes: increased Drp-1 levels (*p* = 0.0049), decreased Opa-1 levels (*p* = 0.0012), and unchanged Mfn-2 levels compared to young controls. In AD, elevated levels of Drp-1 (*p* = 0.0362), Opa-1 (*p* ≤ 0.0001), and Mfn-2 (*p* ≤ 0.0001) are observed compared to aging cases (Figure 5A–C).

Hippocampal aging in CA2 shows persistent Drp-1 level elevation (*p* ≤ 0.0001) with a minor downward trend in Opa-1 (*p* = 0.1093) levels and unchanged Mfn-2 levels compared to young controls. In AD, Opa-1 levels demonstrate a decreasing trend (*p* = 0.0730) while Drp-1 shows an increasing tendency (*p* = 0.3236), though without statistical significance, associated with markedly elevated Mfn-2 levels (*p* ≤ 0.0001) compared to aging cases (Figure 5D–F).

### 2.5. Basal Nuclei (Head of the Caudate Nucleus)

The most numerous medium spiny neurons in the caudate nucleus head region were evaluated. Immunohistochemical staining of all three studied markers in perikarya showed diffuse and relatively homogeneous patterns.

**Figure 6:**
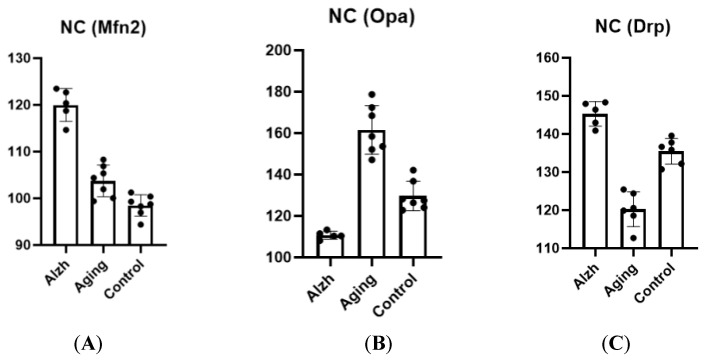
Results of morphometric analysis for immunohistochemical distribution of Mfn-2 (**A**), Opa-1 (**B**), and Drp-1 (**C**) markers in neurons of the caudate nucleus head. Ordinate axis—arbitary unit.

Aging was associated with decreased Drp-1 levels (*p* ≤ 0.0001), increased Opa-1 levels (*p* ≤ 0.0001), and elevated Mfn-2 levels (*p* = 0.0053) in the caudate nucleus head compared to controls (young). In AD, the caudate nucleus head demonstrated elevated Drp-1 (*p* ≤ 0.0001) and Mfn-2 (*p* ≤ 0.0001) levels, and decreased Opa-1 levels (*p* ≤ 0.0001) compared to aging cases (Figure 6).

### 2.6. β-Amyloid

**Figure 7:**
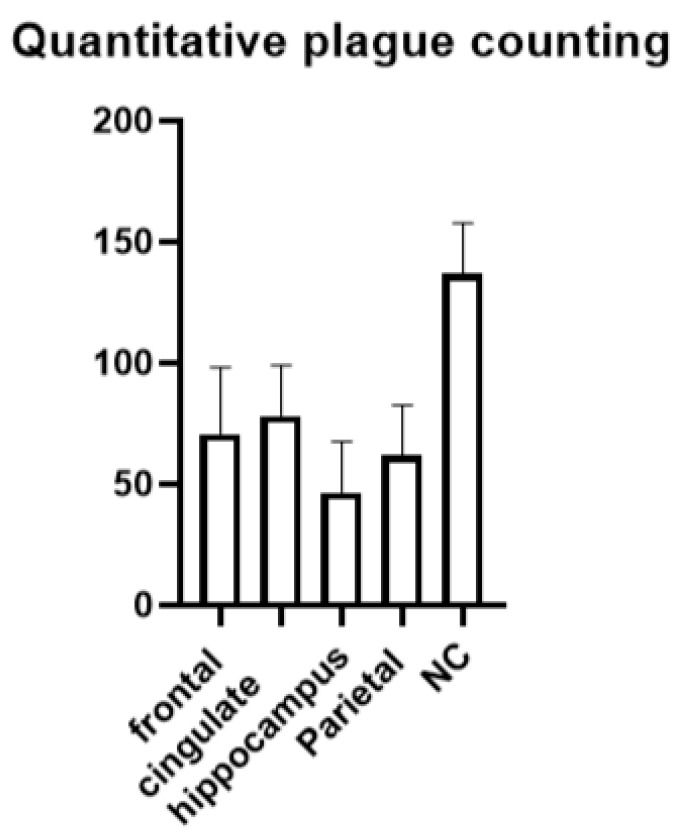
Results of morphometric analysis for amyloid plaque counts in various brain regions in AD. NC—nucleus caudatus. Ordinate axis—arbitary unit.

The distribution of amyloid plaques in AD revealed the following patterns: the middle frontal gyrus, anterior cingulate gyrus, and inferior parietal lobule showed no differences in β-amyloid deposition area or plaque count. The hippocampal region exhibited relatively smaller β-amyloid deposition areas and fewer plaques compared to other studied cortical structures. However, the basal ganglia region showed the largest area occupied by β-amyloid plaques (Figure 7).

## 3. Discussion

### 3.1. Changes in Mitochondrial Dynamics in Alzheimer’s Disease and Normal Aging

Our findings support the concept of heterogeneity in the variants of pathological changes in AD, both in different brain regions and in various areas within a single region [26,27,28].

**Figure 8:**
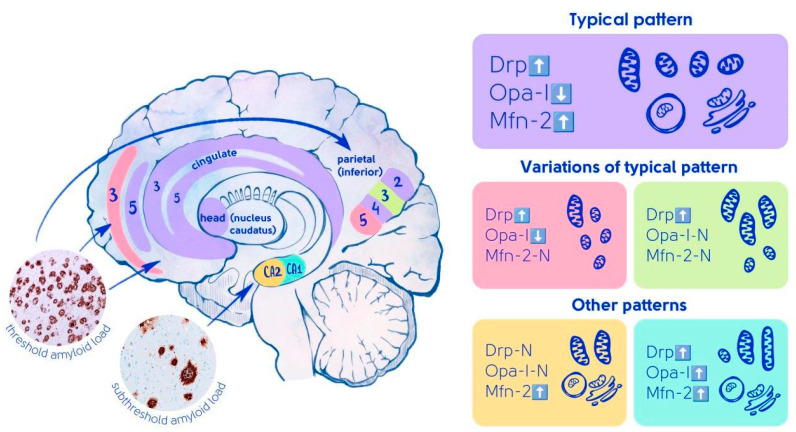
Various patterns of mitochondrial dynamics alterations in neurons characteristic of patients with AD. The colors indicate the patterns: purple color represents a typical pattern, indicating an increase in Drp-1 (up arrow), a decrease in Opa-1 (down arrow), and an increase in Mfn-2 (up arrow); pink color represents a variant of the typical pattern, including an increase in Drp-1 (up arrow), a decrease in Opa-1 (down arrow), and an unchanged amount of Mfn-2 (labeled “N”); green color represents a variant of the typical pattern, including an increase in Drp-1 (up arrow), no change in Opa-1 (labeled “N”), and no change in Mfn-2 (labeled “N”).Other patterns are indicated by the following colors: yellow indicates a pattern that includes unchanged levels of Drp-1 (labeled “N”) and Opa-1 (labeled “N”), and increased levels of Mfn-2 (arrow pointing up); blue indicates a pattern characterized by increases in all proteins studied: Opa-1, Drp-1, and Mfn-2 (arrows pointing up). In the illustration of the sagittal brain section, the zones are labeled, and the areas of interest are indicated: layer 3, layer 5, and the CA2 and CA2 zones of the hippocampus.

When comparing the immunohistochemical markers used in the brain in AD with those in normal aging, we identified a typical pattern of changes characteristic of AD (Figure 8), observed in most of the studied regions and areas, characterized by an increase in the levels of Drp-1 and Mfn-2 markers and a decrease in the intensity of the Opa-1 marker (Figure 1), which causes a shift in mitochondrial dynamics towards mitochondrial fission (increase in Drp-1 marker) and apparently leads to increased organelle fragmentation and greater susceptibility to oxidative stress [29]. Deficiency of the Opa-1 protein leads not only to reduced mitochondrial fusion but also to decreased functional activity of the electron transport chain, as well as to disruption of the morphology of mitochondrial cristae, whose normal state is necessary for maintaining the organelle’s viability [14,30,31]. A potential mechanism of neuronal adaptation to such pathological conditions may involve an increase in the Mfn-2 protein, which, in addition to activating the fusion of outer mitochondrial membranes, facilitates the formation of mitochondria-endoplasmic reticulum contact sites through mitochondria-associated membranes (MAM). These complexes regulate calcium influx into mitochondria, the permeability of the outer mitochondrial membrane, and improve mitochondrial membrane potential [32,33,34]. Furthermore, the compensatory nature of increased Mfn-2 levels may be associated with its role in selective mitophagy and neuronal transport processes [35,36,37]. Thus, the characteristic pattern of mitochondrial dynamics alteration in AD is marked by a shift in mitochondrial dynamics towards fission and enhanced mitochondrial fragmentation, leading to destabilization of organelle membranes and compensation of increased Mfn-2 protein, which appears to enhance endoplasmic reticulum contact with moderately damaged mitochondria while directing irreversibly damaged mitochondria towards selective mitophagy.

We identified two additional patterns of mitochondrial dynamics alterations where the level of the Mfn-2 marker remained unchanged. The first pattern was characterized by an increase in Drp-1 levels accompanied by a decrease in Opa-1 levels, while the second pattern showed an increase in Drp-1 levels with unchanged Opa-1 levels. The absence of changes in Mfn-2 levels may be attributed to less pronounced cristae damage and a relatively lower level of oxidative stress, which does not require activation of Mfn-2-dependent compensatory mechanisms. Thus, all three mitochondrial dynamics alteration patterns are characterized by intensified mitochondrial fission. A prolonged shift in the balance towards mitochondrial fission may lead to destabilization of mitochondrial membranes, reduced functional activity of the electron transport chain, and increased oxidative stress, which in turn activates compensatory responses aimed at enhancing neuroprotection. Based on this, we hypothesize that the latter two patterns represent subtypes of the standard pattern of reactive mitochondrial dynamics alterations and may be explained by variations in functional load or its redistribution depending on the extent of damage in AD.

A distinct pattern of altered mitochondrial dynamics in AD has been identified in the hippocampus. Specifically, neurons in the CA1 region demonstrate increased levels of all three studied markers, while neurons in the CA2 region show only a trend toward elevated Drp-1 (*p* = 0.3236) marker levels and reduced Opa-1 (*p* = 0.0730), though a statistically significant increase in Mfn-2 marker levels is observed. This suggests that even with a tendency toward mitochondrial fission dominance, CA2 neurons already exhibit a compensatory response through increased Mfn-2 marker levels, activating all previously described processes. These findings imply that CA2 neurons are most sensitive to mitochondrial dynamic changes, where even minor shifts toward fragmentation trigger neuroprotective compensatory mechanisms. The absence of imbalance between mitochondrial fission and fusion in CA1 neurons may be explained by enhanced fragmentation of irreversibly damaged mitochondria targeted for selective mitophagy and fusion of less compromised mitochondria with increased endoplasmic reticulum contact sites to optimize mitochondrial membrane functionality.

Changes in mitochondrial dynamics during normal aging compared to their characteristics in the young brain were more heterogeneous in the studied brain regions. Specifically, neurons in the head of the caudate nucleus exhibited a pronounced tendency toward intensified mitochondrial fusion alongside reduced activity of the organelle fission. However, neurons in the anterior cingulate cortex showed an overall decrease in mitochondrial dynamics activity, encompassing both mitochondrial fission and fusion. Conversely, neurons in the frontal cortex, parietal cortex, and hippocampus demonstrated a predominance of mitochondrial fission over fusion during aging. The absence of changes in Mfn-2 marker levels in neurons of the studied cerebral cortex regions (along with its reduction in layer V neurons of the frontal cortex) suggests a lack of compensatory activation of Mfn-2-mediated neuroprotective mechanisms. This may be associated with less pronounced neuronal damage compared to AD, despite the apparent similarity in the direction of mitochondrial dynamics shifts toward organelle fission.

Characteristic pattern of increased Drp-1 levels and decreased Opa-1 levels aligns with data obtained from AD models in the study by Manczak et al. [22], indicating activation of mitochondrial fragmentation due to impaired fusion. However, the observed increase in Mfn2 levels both as part of the typical pattern and as an isolated response in the CA2 region of the hippocampus contradicts this study but corresponds to findings by Xu et al. [24] on elevated Mfn2 levels in APP mice. Furthermore, our study revealed that the CA1 hippocampal region exhibited increased levels of all three investigated markers, which also aligns with data from Xu et al. [24]. Thus, our results demonstrate that the imbalance in mitochondrial dynamics is not limited to a unidirectional shift toward fission or fusion but represents a complex, potentially compensatory or stage-dependent restructuring of processes requiring further investigation in distinct brain regions (Table 1).

Additionally, our findings may indicate a fundamental difference between alterations in mitochondrial dynamics in AD and those occurring during physiological aging.

Alterations in mitochondrial dynamics in neurons in AD may be partially associated with neuronal responses to extracellular tissue factors, including amyloid deposition. Typical patterns of mitochondrial dynamics changes in AD coincides with an increase in level of β-amyloid load, as observed in the middle frontal gyrus, anterior cingulate gyrus, and inferior parietal lobule. A hippocampus-specific pattern of mitochondrial dynamics alterations was noted with the lowest β-amyloid plaque deposition. These data indicate a distinct mechanism of damage in hippocampal regions.

### 3.2. Regional Specificities of Mitochondrial Dynamics Alterations in AD and Aging

The typical pattern of mitochondrial dynamics alterations in AD described above was observed in neurons of the caudate nucleus head, layers 3 and 5 of the anterior cingulate gyrus, layers 2 and 4 of the inferior parietal lobule, and layer 5 of the frontal cortex. Changes in mitochondrial dynamics similar to the typical pattern were noted in neurons of layers 3 and 5 of the inferior parietal lobule, as well as in neurons of layer 3 of the frontal cortex. The variability of alterations may be associated with differing degrees of regional damage, redistribution and reduction of functional load on neurons in affected areas, as well as varying severity of neuronal compensatory potential in AD.

As previously stated, neurons in the CA1 and CA2 hippocampal regions demonstrated a distinct pattern of mitochondrial dynamics alterations in AD. In normal aging, we observe a pronounced shift toward mitochondrial fission, whereas in AD this does not occur—instead, either a tendency toward fission or concurrent activation of both fission and fusion is observed. Thus, the alterations detected in hippocampal neurons may reflect a unique nature of reactive changes in this region during AD progression.

## 4. Materials and Methods

The study was conducted at the Neuropathology Laboratory of the Russian Center of Neurology and Neurosciences, Moscow, Russia.

The autopsy material from AD patients was obtained from 10 deceased individuals with confirmed Alzheimer’s disease (Braak stages 3–4) aged over 85 years (85–89 years), whose cause of death was pulmonary thromboembolism in the presence of post-infarction or atherosclerotic cardiosclerosis associated with hypertension. For comparative analysis, we used autopsy material from two control groups: the first group—normal aging, material obtained from individuals over 85 years (85–99 years) without Alzheimer’s disease who died from similar causes; the second group—young patients aged 35–45 years diagnosed with sudden cardiac death. Exclusion criteria (for all three groups): acute and previous stroke, neuromuscular diseases, cardiovascular and other systemic diseases with decompensation (severe insufficiency), acute infectious, infectious-allergic and autoimmune diseases, malignant tumors of any localization at stages III–IV, alcohol and drugs misuse. Additionally, the study excluded deceased individuals with asymptomatic hemodynamically significant atherosclerotic stenosis of cerebral arteries (arterial lumen narrowing ≥ 70%), as well as those with a history of grade 2–3 arterial hypertension. For microscopic examination in each case, tissue blocks measuring 2–2.5 cm in length, 1–1.5 cm in width, and 0.5–0.7 cm in thickness were excised from the following brain regions of pre-fixed specimens: anterior third of the middle frontal gyrus, anterior cingulate gyrus, middle part of the caudate nucleus head, hippocampus from the middle temporal lobe, and upper part of the inferior parietal lobule. The excised blocks were fixed in 10% neutral buffered formalin solution for 24 h, followed by standard processing (alcohol dehydration, xylene treatment) and paraffin embedding.

For immunohistochemical studies, 3 μm thick sections were prepared using a microtome, mounted on SuperFrost Plus positively charged slides (Thermo Scientific, Waltham, MA, USA), and dried in a thermostat for 24 h at 48 °C. For general observation purposes, the obtained histological specimens were stained with hematoxylin and eosin (H&E) and using Nissl staining. The following set of four antibodies was used for immunohistochemical analysis:

1. Anti-β-Amyloid Antibody (1–42); polyclonal; GTX134510; GeneTex (Zeeland, MI, USA); dilution 1:500;
2. Mfn-2 Polyclonal Antibody; polyclonal; ThermoFisher (Waltham, MA, USA); PA5-89321; dilution 1:200;
3. Opa-1 Monoclonal Antibody (1E8-1D9); monoclonal; Invitrogen (Carlsbad, CA, USA); MA5-16149; dilution 1:200;
4. Anti-Drp-1 antibody; monoclonal; Abcam (Waltham, MA, USA); ab184247; dilution 1:200.

To detect antibody binding, the Novolink Polymer Detection System (Leica Biosystems, Nussloch, Germany) reagent kit was used for polymer-based visualization with peroxidase.

Serial sections were cut from paraffin blocks. Each antibody (Drp-1, Mfn-2, Opa-1) was applied to a separate section using chromogenic immunohistochemistry. All three serial sections were then compared.

Microphotographs of specimens obtained using a Leica DMLB microscope and Leica DC-300 digital camera (for photometric applications) were analyzed with Leica Qwin 2.7 software.

For analysis, 150 neurons with a distinguishable nucleus and nucleolus were randomly selected from 10 to 12 visual fields from the corresponding layer of each brain region studied (layers 3 and 5 of the frontal cortex, layers 3 and 5 of the cingulate cortex, basal nuclei regions, hippocampal CA1 and CA2 areas, and layers 2, 3, 4, and 5 of the parietal cortex), stained with DAB and hematoxylin. All donors contributed equally to the quantitative assessment, after which mean values for each marker were obtained for each donor; for group comparisons, the donor was used as the true unit of analysis.

Neuronal cell bodies and nuclei were outlined using a Wacom graphic tablet, excluding nuclear areas. The staining density of selected regions was quantified in arbitrary units (256 grayscale levels of 8-bit images, where 0 represents white and 255 represents black), with background staining values subtracted.

Statistical analysis and data visualization were performed using Statistica 13.0 and GraphPad Prism 8.0 software. Normality of distribution was assessed using the Shapiro–Wilk test. Intergroup comparisons were made using one-way ANOVA with Fisher’s LSD post hoc test. Differences were considered statistically significant at *p* < 0.05.

## 5. Conclusions

Our findings suggest specific patterns of metabolic process alterations in neurons, which are not limited to a single brain region but involve multiple areas and exhibit a uniform pattern. Specific patterns may be associated not only with the disease stage but also with the multifactorial nature of neuropathological changes in brain tissue in AD.

Investigating mitochondrial dynamics changes across different brain regions in AD may form the basis for developing targeted AD therapies aimed at enhancing compensatory neuroprotection in the most damaged or vulnerable brain areas. The identified regional heterogeneity of neuronal responses must be considered when using targeted pharmacological agents, including those intended to modulate mitochondrial dynamics.

## Figures and Tables

**Table 1:** Summary of changes in different areas of the brain in Alzheimer’s disease.

Zone	DRP-1	Opa-1	Mfn-2	Amyloid Load	Research by Other Authors
Middle frontal gyrus, 3 layer	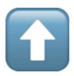	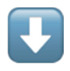	Normal	+++	The studies are presented to a greater extent in various areas of the hippocampus, less in the frontal cortex, parietal and cingulate, and are practically absent in the head of the caudate nucleus: Increase DRP-1 [22,38] Decrease Opa-1 [23,38,39,40] Increase MFN2 [23,39,41]
Middle frontal gyrus, 5 layer	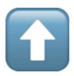	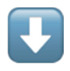	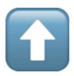		
Anterior cingulate gyrus, 3 layer	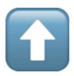	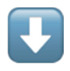	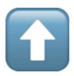	+++	
Anterior cingulate gyrus, 5 layer	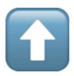	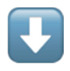	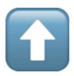		
Head of the caudate nucleus	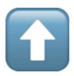	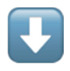	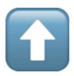	++++	
Hippocampus, CA1	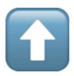	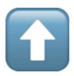	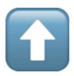	+	
Hippocampus, CA2	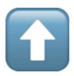 (tend)	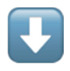 (tend)	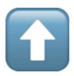		
inferior parietal lobule, 2 layer	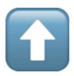	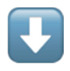	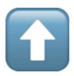	++	
inferior parietal lobule, 3 layer	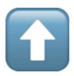	Normal	Normal		
inferior parietal lobule, 4 layer	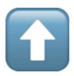	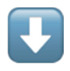	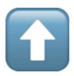		
inferior parietal lobule, 5 layer	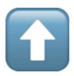	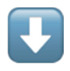	Normal		

The table shows a visual representation of the studied proteins’ changes by using graphical symbols: up arrow—increase compared to the aging group; down arrow—decrease compared to the aging group; Normal—no significant changes compared to the aging group. Amyloid load is visualized with a “+”: the amount of + represents a quantitative assessment of amyloid deposition with “+”—light load; “++++”—severe load.

## Data Availability

All the data are shown in the main manuscript.
